# Recurrence patterns and lifetime performance of parity 1 sows in breeding herds with different weaning-to-first-mating intervals

**DOI:** 10.1186/s40813-019-0122-0

**Published:** 2019-06-28

**Authors:** Yu Yatabe, Ryosuke Iida, Carlos Piñeiro, Yuzo Koketsu

**Affiliations:** 1 School of Agriculture, Meiji University, Higashi-mita 1-1-1, Tama-ku, Kawasaki, Kanagawa 214-8571 Japan; 2 PigCHAMP Pro Europa S.L., c/Calle Dámaso Alonso, 14, 40006 Segovia, Spain

**Keywords:** Cohort study, Farm management, Lifetime performance, Primiparous sows, Swine, Weaning-to-first-service interval

## Abstract

**Background:**

Our objectives were 1) to compare reproductive performance across parities and lifetime performance of parity 1 sows in six weaning-to-first-mating interval groups (WMI 0–3, 4, 5, 6, 7–20 and 21 days or more), 2) to determine the recurrence patterns and repeatability of WMI, and 3) to quantify factors associated with the probability of parity 1 sows having WMI 4 days. Examined data comprised 691,276 parity and 144,052 lifetime records of sows in 155 Spanish herds, served between 2011 and 2016. Mixed-effects models were applied to the data. Variance components analysis determined WMI repeatability.

**Results:**

Proportions of parity 1 sows with WMI 0–3, 4, 5, 6, 7–20 and 21 days or more were 4.1, 30.0, 38.4, 7.9, 12.7 and 6.9%, respectively. Of the parity 1 sows with WMI 0–4 days, 43.3–60.5% had WMI 4 days in later parities, whereas 33.9–48.9% of those with WMI ≥5 days had WMI 5 days; WMI repeatability was 0.11. Parity 1 sows with WMI 4 or 5 days had 0.3–2.1 days shorter WMI in later parities than those with WMI ≥7 days (*P* <  0.05). Parity 1 sows with WMI 4 or 5 days also had 0.6–2.1 more annualized lifetime piglets born alive than those with WMI ≥7 days (*P* <  0.05). Notably, parity 1 sows with WMI 4 days had 0.3 more annualized lifetime piglets born alive than those with WMI 5 days (*P* <  0.05).

**Conclusion:**

The WMI in parity 1 could be a useful predictor for subsequent reproductive performance and lifetime performance of sows.

## Background

Weaning-to-first-mating interval (WMI) is one of the key performance indicators for sow productivity in breeding herds, and is a major part of non-productive days of sows [1]. Approximately 90% of sows have WMI of 0–6 days [2], but WMI of the other 10% of sows can vary widely. A study in Thailand has shown that parity 1 crossbred sows with WMI 0–5 days had greater longevity and more lifetime piglets born alive than those with WMI 6 days or more [3]. Another study has shown that sows with WMI 4–6 days had higher farrowing rates than those with WMI 7–20 days [4]. Also, it is reported that purebred Hampshire sows with WMI 4 days had higher farrowing rates than those with WMI 5 or 6 days [5]. However, no single study has compared the difference in sow reproductive performance between parity 1 sows in six WMI groups, namely WMI 0–3, 4, 5, 6, 7–20 and 21 days or more.

A study in Japan reported that more than 85% of sows with WMI 4–6 days in parity 1 also had WMI 4–6 days in parity 2 [4]. However, there have not been any studies in breeding herds about recurrence patterns of WMI in later parities nor the repeatability of WMI. Furthermore, even though prolonged WMI is known to be associated with shorter lactation length [6] and higher numbers of piglets weaned [7], there have not been any reports about the effects of lactation length or the number of piglets weaned on the probability of parity 1 sows having a certain WMI.

Therefore, the objectives of the current study were 1) to compare subsequent reproductive performance across parities and lifetime performance in six WMI groups of parity 1 sows, 2) to assess the recurrence patterns and repeatability of WMI and 3) to quantify factors associated with the probability of parity 1 sows having WMI 4 days.

## Methods

### Studied herds

A veterinary consultancy firm (PigCHAMP pro Europa S.L., Segovia, Spain) requested all client producers to mail their data files on a regular basis to build up a sow database. In July 2017, by-parity reproductive performance and lifetime performance records of sows in 155 Spanish herds, which allowed their data to be used for research, were extracted from the database.

Overall mean herd size in Spain in December 2013 was 131 sows, estimated by dividing the 2,568,450 recorded sows by the 19,630 breeding herds [8]. In the present study, mean herd size (± SEM) in our studied herds during 2016 was 913 ± 60.1 sows with a range between 87 and 5640 sows. Also, the herd mean of the number of piglets weaned per sow per year (± SEM) in these studied herds was 26.3 ± 0.19 piglets with a range between 19.6 and 33.3 piglets. The lactation and gestation diets of sows in the studied herds were formulated using cereals (barley, wheat and corn) and soybean meal. In the studied herds, sows were mainly crossbreds between Landrace and Large White, and replacement gilts were either purchased from international breeding companies or home-produced through internal multiplication programs.

### Study design, data and exclusion criteria

The present study was designed as a retrospective cohort study utilizing by-parity service records and subsequent reproductive records, from first-service in parity 1 to removal, for 150,565 sows entered between 2011 and 2013, and removed between 2011 and 2017. The data comprised 728,928 parity records of sows serviced from January 2011 to December 2016. When the data were collected, 2762 (1.8%) sows had no record of parity at removal, so these sow records were excluded.

Sow records were also excluded if lifetime non-productive days were 294 days or more (1530 sows; 99th percentile; [9]). Further records of sows were excluded if the parity records of a sow met any of the following criteria in their lifetime: total number of piglets born was either 0 or 31 piglets or more (681 sows; [10]) and WMI was 61 days or more (1540 sows; [11]). Hence, the final dataset comprised 691,276 parity records and 144,052 lifetime records for sows that had at least one WMI. Also, when analyzing the probability of parity 1 sows having WMI 4 days, additional exclusions were made for records with 0 piglets weaned (973 records), lactation length of 0–9 days or 42 days or more (1608 records) and age at first-mating (17,454 records; [12]).

### Categories and definitions

Sows were categorized into six WMI groups based on WMI in parity 1: WMI 0–3, 4, 5, 6, 7–20 and 21 days or more. The categorization was based on previous reports; 0–3 days [13], 4, 5, 6 days [5], 7–20 days [4], and 21 days or more [14]. In addition, we have hypothesized that there are differences in subsequent reproductive performance and lifetime performance between parity 1 sows with WMI 4, 5 and 6 days, because it is reported that LH patterns differ between sows with WMI 3–4 days and 5 days or more [15]. Means (± SEM) of WMI 0–3, 7–20 and 21 days or more groups were 2.1 ± 0.02, 11.2 ± 0.03 and 31.1 ± 0.09 days, respectively. Also, there were five parity groups: parity 1, 2, 3, 4 and 5 or higher.

Lifetime piglets born alive was the sum of the number of piglets born alive in a sow’s lifetime. Annualized lifetime piglets born alive was calculated as the lifetime piglets born alive divided by the sum of reproductive herd life days × 365. The reproductive herd life days was defined as the number of days from the date that a gilt was first-served to its removal date. Lifetime non-productive days was defined as the number of days when a sow was neither gestating nor lactating during its reproductive herd life.

### Statistical analysis

All analyses were conducted using SAS University Edition (SAS Inst. Inc., Cary, NC). A chi-square test was used to compare proportions (%) of WMI groups between parity 1 sows and sows in parity 2 or higher.

Three statistical models were created. Model 1 analyzed parity records to compare the WMI groups for subsequent reproductive performance. The Model 1 was constructed by applying a 3-level liner mixed-effects model using the MIXED procedure for a continuous outcome, and by applying a 3-level mixed-effects logistic regression model using the GLIMMIX procedure for farrowing rate or probability of parity 1 sows having a WMI 4 days. Model 1 was also used to account for the clustering of sows within a herd (random statement) and the correlation between repeated measures in the same sows (MIXED, repeated statement; GLIMMIX, random_residual_statement). The model included the following factors as fixed effects: the WMI groups, parity groups, entry year and two-way interactions between the WMI groups and parity groups. When the model assessed piglets born alive, it also included previous quarterly service seasons, whereas when it assessed WMI and farrowing rate, it included quarterly farrowing seasons. Quarterly seasons were January–March, April–June, July–September and October–December.

Model 2 was applied to compare the WMI groups for lifetime performance, and Model 3 quantified factors associated with the probability of parity 1 sows having WMI 4 days, respectively. A 2-level liner mixed-effects model was applied to Model 2 using the MIXED procedure to account for the clustering of sows within a herd (random statement). The following factors were included as fixed effects in Model 2 for lifetime performance: the WMI groups, quarterly herd entry seasons and entry year. In Models 1 and 2, pairwise multiple comparisons were performed using the Tukey-Kramer test. In addition, a 2-level mixed-effects logistic regression model was applied to Model 3 using the GLIMMIX procedure to account for the clustering of sows within a herd (GLIMMIX, random statement). Also, age at first-mating was included as a covariate in Models 1 and 2.

Model 3 included lactation length, piglets weaned, age at first-mating, quarterly farrowing seasons and entry year as fixed effects. Also, included were the quadratic expressions of lactation length, piglets weaned and age at first-mating, and the two-way interactions between lactation length and piglets weaned. Lactation length,piglets weaned and age at first-mating were centered at the grand mean value. For all analyses, the significance level was set at 0.05.

### Repeatability of WMI and intraclass correlation coefficients

Variance components analysis was conducted using the VARCOMP procedure. Because the SAS software could not handle more than 5000 sows for the analysis, 5000 sows were randomly selected from the dataset with the SURVEYSELECT procedure. Repeatability for WMI was determined by the following equation [16]:

$$ \mathrm{Repeatability}\kern0.5em =\kern0.5em \left({\sigma}_v^2+{\sigma}_u^2\right)/\left({\sigma}_v^2+{\sigma}_u^2+{\sigma}_{\varepsilon}^2\right), $$

in which ${\sigma}_v^2$ is the between-herd variance, ${\sigma}_u^2$ is the between-sow variance and ${\sigma}_{\varepsilon}^2$ is the variance at the individual record level. The model for WMI included parity groups, quarterly farrowing month groups and entry year as fixed effects, and also herds and sows nested within a herd as random effects.

The intraclass correlation coefficients (ICC) were calculated by the following equation [17] to assess the variation in the amount of the probability of parity 1 sows having WMI 4 days that could be explained by the herd:

$$ \mathrm{ICC}\left(\mathrm{individualrecordswithinthesameherd}\right)\kern0.5em =\kern0.5em {\sigma}_v^2/\left({\sigma}_v^2+{\pi}^2/3\right), $$

in which ${\sigma}_v^2$ is the between-herd variance and *π*^2^/3 is the assumed variance at the individual record level.

## Results

Mean WMI (± SEM) was 5.9 ± 0.01 days (Table 1). The proportions of parity 1 sows with WMI 0–3, 4, 5, 6, 7–20 and 21 days or more were 4.1, 30.0, 38.4, 7.9, 12.7 and 6.9%, respectively (Table 2). There was a difference in the proportions in WMI between parity 1 sows and sows in parity 2 or higher (*P* <  0.05). Parity 1 sows had higher proportions of WMI 5 days or more than those in parity 2 or higher.

**Table 1:** Reproductive performance of sows in 155 herds

Measurements	*n*	Mean	SEM	Median (IQR)
Lifetime records				
Age at first-mating	126,598	254.4	0.11	249 (235–273)
Number of parity at removal	144,052	5.5	0.01	6 (4–7)
Reproductive herd life days	144,052	834.1	0.86	882 (573–1098)
Lifetime piglets born alive	144,052	68.0	0.09	71 (42–93)
Lifetime non-productive days	144,052	74.1	0.14	57 (34–101)
Annualized lifetime piglets born alive	144,052	28.7	0.02	29 (25–33)
Annualized lifetime piglets weaned	144,052	25.4	0.01	26 (23–28)
Parity records				
Served parity	691,276	3.4	0.01	3 (2–5)
Weaning-to-first-mating interval, days	691,276	5.9	0.01	5 (4–5)
Farrowing rate, %	691,276	87.5	0.04	–
Number of subsequent piglets born alive	647,814	12.5	0.01	13 (11–15)
Parity 1 records				
Lactation length, days^a^	141,471	23.8	0.01	23 (21–26)
Number of piglets weaned^a^	141,471	11.0	0.01	11 (10–12)
Weaning-to-first-mating interval, days	144,052	7.3	0.01	5 (4–6)

*SEM* standard error of the mean, *IQR* interquartile range

^a^The remaining records (144,052 - *n*) were regarded as missing records

**Table 2:** Relative frequency distributions (%) of weaning-to-first-mating interval (WMI) in parity 1 sows (144,052 records) and parity 2 or higher sows (547,224 records) categorized in six WMI groups^a^

WMI groups (days)	Parity 1		Parity 2 or higher		Chi-square test
	*n*	%	*n*	%	
0–3	5909	4.1	46,431	8.5	*P* < 0.05
4	43,178	30.0	243,114	44.4	
5	55,378	38.4	179,095	32.7	
6	11,377	7.9	24,506	4.5	
7–20	18,219	12.7	39,944	7.3	
21 or more	9991	6.9	14,134	2.6	

^a^Frequency within a column totals 100%

Table 3 shows the WMI recurrence patterns in each WMI group in parity 1. For example, 60.5% of the parity 1 sows with WMI 4 days had the same WMI (4 days) in parity 2. Also, 43.3% of the parity 1 sows with WMI 0–3 days had WMI 4 days in parity 3. Overall, 43.3–60.5% of the parity 1 sows with WMI 0–4 days had WMI 4 days in later parities, whereas 33.9–48.9% of the parity 1 sows with WMI 5 days or more had WMI 5 days in later parities. Furthermore, looking only at parity 1 sows with WMI 0–3 days, 21.4–24.0% had WMI 0–3 days in later parities. Also, looking only at parity 1 sows with WMI 7–20 days, 9.4–14.6% had WMI 7–20 days in later parities. The repeatability of WMI was 0.11 (Table 4).

**Table 3:** Cross-classified relative frequency distributions (%) of six weaning-to-first-mating interval (WMI) groups in parity 1 and subsequent parities^a^

WMI groups in parity 1 (days)	Weaning-to-first-mating interval in subsequent parities (days)					
	0–3	4	5	6	7–20	21 or more
	Percentages of sows (%)					
Parity 2						
0–3	21.4	45.5	19.3	2.9	8.1	2.8
4	9.1	60.5	19.3	2.7	6.3	2.1
5	3.9	32.2	48.9	5.6	6.7	2.7
6	3.0	25.8	46.5	11.3	9.5	3.9
7–20	5.1	31.5	36.2	8.4	14.6	4.2
21 or more	4.2	28.6	38.4	8.0	11.5	9.3
Parity 3						
0–3	24.0	43.3	17.1	2.9	10.3	2.4
4	12.0	60.4	16.8	2.1	6.7	2.0
5	5.3	38.1	43.7	4.3	6.3	2.3
6	4.2	30.6	44.4	9.2	8.4	3.2
7–20	6.0	33.7	35.9	7.5	13.1	3.8
21 or more	4.9	29.4	36.6	8.6	12.0	8.5
Parity 4						
0–3	22.0	46.2	17.7	2.5	9.2	2.4
4	11.7	60.4	17.6	2.2	6.3	1.8
5	6.1	40.2	40.9	4.1	6.3	2.4
6	4.6	32.0	44.9	7.5	8.0	3.0
7–20	7.1	37.9	33.9	6.9	11.0	3.2
21 or more	6.5	35.5	36.0	6.9	9.9	5.2
Parity 5						
0–3	23.3	46.0	17.0	2.5	9.1	2.1
4	13.8	59.0	16.3	2.2	6.7	2.0
5	7.5	42.9	37.9	3.8	5.8	2.1
6	5.7	36.9	40.7	7.4	6.6	2.7
7–20	7.7	40.3	33.9	5.5	10.1	2.5
21 or more	7.2	37.2	35.9	6.9	8.3	4.5
Parity 6 or higher						
0–3	22.7	46.9	19.0	2.4	7.2	1.8
4	13.4	59.1	18.4	2.0	5.4	1.7
5	7.9	42.9	38.8	3.8	4.9	1.7
6	6.0	35.1	42.7	7.2	6.8	2.2
7–20	8.4	39.1	34.9	5.9	9.4	2.3
21 or more	8.0	37.5	36.2	6.5	8.1	3.7

^a^Frequency within a row totals 100%

**Table 4:** Repeatability of weaning-to-first-mating interval (days)

Variances				
Sow	Herd	Error	Total	Repeatability
0.76	2.60	26.96	30.32	0.11

There were significant main effects of the WMI groups and parity groups, and also two-way interactions between these two groups for farrowing rates, subsequent piglets born alive and subsequent WMI (*P* <  0.05). Parity 1 sows with WMI 4 or 5 days had 0.3–2.1 days shorter subsequent WMI in later parities than parity 1 sows with WMI 7 days or more (Table 5; *P* <  0.05). Also, parity 1 sows with WMI 4 days had 1.0% higher farrowing rates in parity 1 than sows with WMI 5 days (*P* <  0.05). Furthermore, they had 0.2 more subsequent piglets born alive in parities 1, than sows with WMI 5 days (*P* <  0.05). Additionally, parity 1 sows with WMI 0–3 days had 4.2–5.2% lower farrowing rates in parity 1 than those with WMI 4 or 5 days (*P* <  0.05).

**Table 5:** Comparisons of farrowing rates, subsequent piglets born alive and subsequent weaning-to-first-mating interval (WMI) of six WMI groups in consecutive served parities^1^

Served parities						
WMI groups in parity 1 (days)	*n* ^*2*^	1	2	3	4	5 or higher
				Mean (± SE)		
Farrowing rates, %						
0**–**3	5909	82.8 (0.65)c,y	87.2 (0.52)ab,wx	87.6 (0.59)ab,w	87.8 (0.57)ab,w	85.1 (0.53)bc,xy
4	43,178	88.0 (0.31)a,w	88.1 (0.32)a,w	88.8 (0.31)a,w	88.0 (0.33)a,w	86.5 (0.33)ab,x
5	55,378	87.0 (0.33)b,y	88.1 (0.31)a,w	88.4 (0.30)ab,w	87.6 (0.33)ab,x	87.2 (0.31)a,xy
6	11,377	82.4 (0.53)c,x	88.6 (0.43)a,w	88.6 (0.44)a,w	87.9 (0.33)ab,w	87.3 (0.40)a,w
7**–**20	18,219	81.2 (0.50)c,x	86.9 (0.41)b,w	86.5 (0.43)b,w	86.4 (0.41)b,w	86.0 (0.39)ab,w
21 or more	9991	81.1 (0.57)c,x	85.5 (0.52)b,w	84.5 (0.56)b,w	85.8 (0.58)b,w	84.6 (0.51)c,w
Subsequent piglets born alive						
0**–**3	5590	12.2 (0.081)b,x	12.7 (0.082)w	12.9 (0.084)w	12.5 (0.085)x	12.0 (0.081)y
4	41,288	12.2 (0.070)b,y	12.8 (0.071)w	12.8 (0.070)w	12.6 (0.071)x	12.1 (0.070)z
5	52,657	12.0 (0.070)c,y	12.7 (0.070)w	12.8 (0.070)w	12.6 (0.070)x	12.1 (0.070)y
6	10,672	11.8 (0.075)d,z	12.6 (0.076)wx	12.8 (0.070)w	12.5 (0.078)x	12.2 (0.075)y
7**–**20	16,885	12.1 (0.073)bc,x	12.6 (0.074)w	12.7 (0.074)w	12.6 (0.075)w	12.1 (0.073)x
21 or more	9084	12.7 (0.077)a,w	12.7 (0.077)w	12.7 (0.079)w	12.5 (0.081)x	12.0 (0.078)y
Subsequent weaning-to-first-mating interval, days						
0**–**3	5317	5.6 (0.09)d,w	5.7 (0.09)de,w	5.5 (0.10)d,w	5.4 (0.10)c,wx	5.2 (0.09)c,x
4	39,517	5.5 (0.06)d,w	5.5 (0.06)e,w	5.4 (0.06)d,w	5.4 (0.07)c,w	5.2 (0.06)c,x
5	50,040	5.9 (0.06)c,w	5.7 (0.06)de,x	5.6 (0.06)cd,x	5.5 (0.06)c,y	5.2 (0.06)c,z
6	10,048	6.4 (0.08)b,w	6.0 (0.08)c,x	5.9 (0.08)bc,xy	5.7 (0.08)bc,y	5.4 (0.08)bc,y
7**–**20	15,940	6.6 (0.07)b,w	6.5 (0.07)b,w	6.1 (0.07)b,x	5.8 (0.08)ab,xy	5.6 (0.07)ab,y
21 or more	8251	7.8 (0.08)a,w	7.6 (0.08)a,w	6.5 (0.09)a,x	6.1 (0.09)a,y	5.8 (0.09)a,y

^1^Means and SE were estimated by using mixed models

^2^*n* represents initial number of sows

^a-e^Different superscripts within a column represent significant differences in means (*P* <  0.05)

^w-z^Different superscripts within a row represent significant differences in means (*P* <  0.05)

With regard to lifetime performance (Table 6), there were associations between WMI groups and sow lifetime performance (*P* <  0.05). Parity 1 sows with WMI 4 or 5 days had 0.2–0.7 higher parities at removal, 2.2–9.0 more lifetime piglets born alive, 2.8–31.7 fewer lifetime non-productive days and 0.4–2.1 more annualized lifetime piglets born alive than parity 1 sows with WMI 6 days or more (*P* <  0.05). Parity 1 sows with WMI 4 days had 0.7 more lifetime piglets born alive and 0.3 more annualized lifetime piglets born alive than parity 1 sows with 5 days WMI; they also had 1.2 days fewer lifetime non-productive days than those with WMI 5 days (*P* <  0.05). Additionally, parity 1 sows with WMI 0–3 days had 0.2 lower parities at removal and 3.8–4.3 fewer lifetime piglets born alive than parity 1 sows with WMI 4 or 5 days.

**Table 6:** Comparisons of lifetime performance of sows between six weaning-to-first-mating interval (WMI) groups^1^

WMI groups in parity 1 (days)	*n*	Parity at removal	Lifetime piglets born alive	Lifetime non-productive days	Annualized lifetime piglets born alive	Annualized lifetime piglets weaned
		Mean (± SE)				
0–3	5909	5.6 (0.07)b	67.0 (0.90)b	76.5 (1.72)de	28.5 (0.21)ab	24.5 (0.17)b
4	43,178	5.8 (0.06)a	71.3 (0.80)a	77.1 (1.59)e	28.7(0.19)a	25.0 (0.16)a
5	55,378	5.8 (0.06)a	70.8 (0.79)a	78.3 (1.58)d	28.4 (0.19)b	24.9 (0.16)a
6	11,377	5.6 (0.06)b	68.6 (0.84)b	81.1 (1.65)c	28.0 (0.20)c	24.7 (0.17)b
7–20	18,219	5.5 (0.06)c	67.1 (0.82)c	88.3 (1.62)b	27.8 (0.20)d	24.3 (0.17)b
21 or more	9991	5.1 (0.06)d	62.3 (0.85)d	108.8 (1.66)a	26.6 (0.20)e	22.9 (0.17)c

^1^Means and SE were estimated by using mixed models

^a-e^Different superscripts within a column represent significant differences in means (*P* < 0.05)

**Fig. 1:**
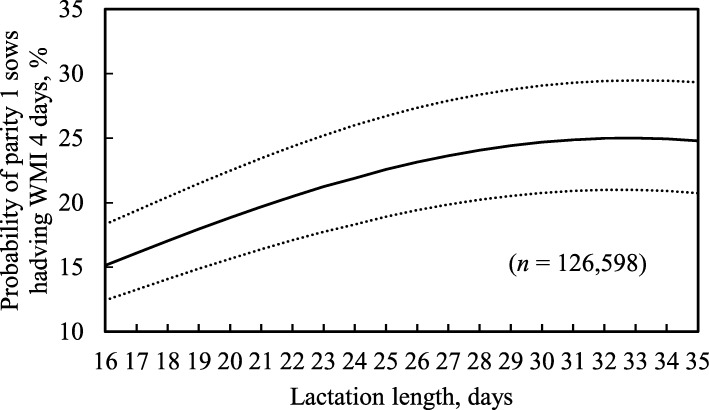
Predicted probability of parity 1 sows having weaning-to-first-mating interval (WMI) 4 days with increasing lactation lengths. Dotted lines show 95% confidence intervals

**Fig. 2:**
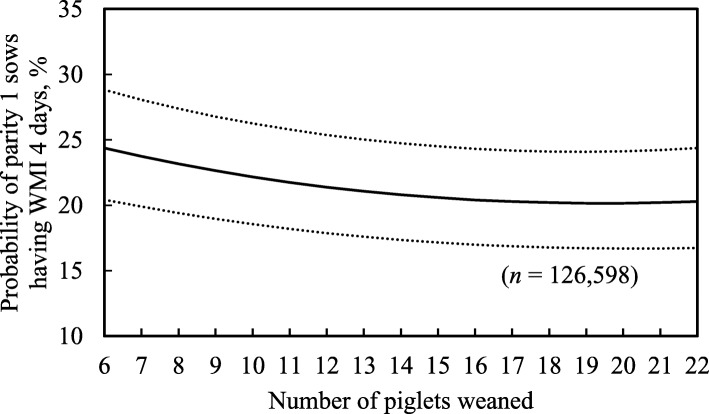
Predicted probability of parity 1 sows having weaning-to-first-mating interval (WMI) 4 days with increasing piglets weaned. Dotted lines show 95% confidence intervals

**Fig. 3:**
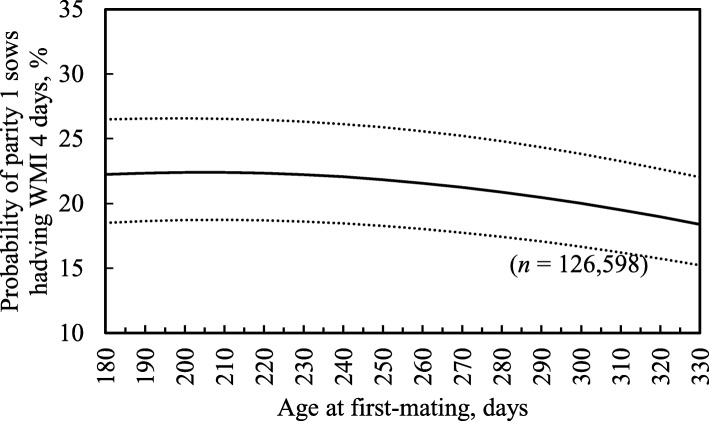
Predicted probability of parity 1 sows having weaning-to-first-mating interval (WMI) of 4 days with different age at first-mating (days). Dotted lines show 95% confidence intervals

Longer lactation length,fewer piglets weaned and lower age at first-matingwere associated with a higher probability of parity 1 sows having WMI 4 days (*P* <  0.05), but there was no such association with the two-way interaction (Table 7; *P* = 0.88). For example, as lactation length increased from 18 to 31 days (5th to 95th percentiles), the probability of parity 1 sows having WMI 4 days increased by 8.2% (Fig. 1). Also, as piglets weaned decreased from 14 to 8 piglets (95th to 5th percentiles), the probability of parity 1 sows having WMI 4 days increased by 2.4% (Fig. 2). Additionally, when age at first-mating decreased from 320 to 220 days, the probability of parity 1 sows having WMI 4 days increased by 4.4% (Fig. 3). However, the probability of parity 1 sows having WMI 4 days did not vary very much between 12 and 22 piglets weaned. With regard to the ICC, the random herd effect explained 34% of total variance values for the probability of parity 1 sows having WMI 4 days.

**Table 7:** Estimates of fixed factors and random effect variance included in the mixed-effects logistic regression model for the probability of parity 1 sows having a weaning-to-first-mating interval (WMI) of 4 days

Fixed and random effects	Probability of parity 1 sows having WMI 4 days	
	Estimate (± SE)	*P*-value
Intercept	- 0.992 (0.1137)	< 0.01
Age at first-mating	- 0.0017 (0.0003)	< 0.01
Age at first-mating squared	0.0002 (0.00004)	< 0.01
Lactation length	0.041 (0.0022)	< 0.01
Lactation length squared	- 0.0023 (0.0003)	< 0.01
Piglets weaned	- 0.023 (0.0039)	< 0.01
Piglets weaned squared	0.0014 (0.0004)	< 0.01
Piglets weaned x lactation length	0.0006 (0.0005)	0.21
Age at first-mating x lactation length	0.0004 (0.00005)	< 0.01
Age at first-mating x piglets weaned	−0.0001 (0.00008)	0.10
Intercept variance at herd level	1.70 (0.22)	–
ICC (records within the same herd), %	34.0	–

*SE* standard error, *ICC* intraclass correlation coefficient

## Discussion

Our study indicated that parity 1 sows with WMI 4 or 5 days had higher lifetime productivity and greater longevity than parity 1 sows with WMI 6 days or more because the sows with WMI 4 or 5 days had shorter WMI and higher farrowing rates in later parities than the parity 1 sow groups with longer WMI. It is possible that the hypothalamic-pituitary-ovary axis of parity 1 sows with WMI 4 or 5 days has greater potential to resume estrus postweaning compared to equivalent sows with WMI 6 days or more. A shorter WMI is strongly related to higher luteinizing hormone concentrations during lactation and postweaning in sows [18, 19]. In addition, our study showed that parity 1 sows with WMI 4 days had the greatest lifetime efficiency and longevity among the six WMI groups.

There was a distinct difference in farrowing rates in later parities between the six WMI groups of parity 1 sows. Low farrowing rates or high farrowing failure are suggested to occur due to decreased GnRH secretion, decreased luteinizing hormone release and impaired corpora lutea functions [20]. It might be possible that there were differences in the potential of the hypothalamic-pituitary-ovary axis between the six WMI groups of parity 1 sows, and that WMI in parity 1 is associated with farrowing rates in later parities.

Our study showed that there was a difference between the six WMI groups in piglets born alive per litter in parities 2 and 3 (0.1–0.2 piglets), and that there were no such differences in parities 4 or higher. This suggests that the WMI in parity 1 is not directly associated with the number of piglets born alive in later parities. Instead, the number of piglets born alive is associated with the number of ovulation and embryo survival [21]. In addition, due to the accumulation of small differences at each parity, and greater longevity, parity 1 sows with WMI 4 or 5 days had more lifetime piglets born alive than those with WMI 6 days or more.

We also found that for parity 1 sows with WMI 0–4 days the most frequent WMI in later parities was WMI 4 days, whereas for sows with WMI 5 days or more in parity 1 the most frequent WMI in later parities was WMI 5 days. These WMI recurrence patterns suggest that parity 1 sows with WMI 0–4 days had a quicker recovery after lactational anestrus than those with WMI 5 days or more. It is hypothesized that parity 1 sows with shorter WMI had gonadotropin secretion characteristics different from those with WMI 5 days or more [15].

Approximately 10% of the parity 1 sows with WMI 7–20 days had WMI 7–20 days in later parities, suggesting that some sows have a weak mechanism for resuming estrus postweaning. Although we do not have data in sows’ appetites or a disease, one possible reason for this is that it is likely that some of the sows had an innately poor appetite, resulting in excessive loss of body weight during lactation and these sows could have the prolonged WMI [3, 22]. Additionally, some parity 1 sows may have a gilt development problem or a feeding problem during gestation [1].

Also, the WMI repeatability of 0.11 in our study was higher than that found in a previous study in Japan, which showed a repeatability of only 0.08 [4]. The reason for the relatively higher repeatability in our study appears that Spanish herds have different management from Japanese herds, such as relating to hormonal treatments or strict culling policy for sows with prolonged WMI.

In addition, the reason for parity 1 sows with WMI 0–3 days continuing to have WMI 0–3 days in subsequent parities could be because some sows have a robust hypothalamic-pituitary-ovary axis function, and so tend to have short WMI in parity 1 and later parities. In our study, the parity 1 sows with WMI 0–3 days had a lower farrowing rate in parity 1 than the sows with WMI 4 or 5 days. It is possible that some of the parity 1 sows with WMI 0–3 days had ovarian cysts and therefore had lower farrowing rates than those with WMI 4 or 5 days. This possibility is supported by a previous study which reported that sows with WMI of only 0–2 days were at high risk of developing cysts which is a cause of low farrowing rates [23]. Also, our study showed that the average parity for removal of parity 1 sows with WMI 0–3 days was lower than that of parity 1 sows with WMI 4 or 5 days. This difference suggests that producers culled more parity 1 sows with 0–3 days than parity 1 sows with 4 or 5 days. However, our study also indicates that the parity 1 sows with WMI 0–3 days were capable of having the same lifetime efficiency as those with WMI 4 or 5 days. Therefore, producers should not hastily cull parity 1 sows with WMI 0–3 days unless they exhibit repeat-breeding or not-in-pig or nymphomania, which are characteristics of ovarian cysts [23–25].

The higher proportions of sows with WMI 5 days or more in parity 1 than sows in parity 2 or higher is consistent with previous studies reporting prolonged WMI in parity 1 sows [26, 27]. This can be explained by the fact that parity 1 sows are still growing and so tend to have immature endocrine systems and low lactation feed consumption [28], which decreases their gonadotropin secretion and slows down ovarian follicle growth in the sows [29].

Our study also found that the probability of parity 1 sows having WMI 4 days was independently associated with increased lactation length,decreased numbers of piglets weaned and decreased ages at first-mating. However, the number of piglets weaned per litter is currently increasing because of genetic improvement [30], so it is not feasible for producers to decrease the number of piglets weaned. Furthermore, our study indicates that the probability of parity 1 sows having WMI 4 days was relatively stable in sows that weaned 12 piglets or more. In addition, increased lactation length simply increases farrowing intervals and decreases sow reproductive efficiency [1], whereas there is EU legisration that reqires weaning age 28 days or higher for piglets [31]. Age at first estrus or first-mating can be decreased by boar exposure [32]. Therefore, in order to increase the probability of sows having WMI 4 days, we recommend using boar exposure to decrease age at-first mating, and also advise increasing feed intake during lactation [33] to meet the increased nutritional need in milk yields for increasing numbers of piglets, because insufficient lactational feed intake is a primary cause of prolonged WMI [28]. Additionally, a development problem or a feeding problem in gestation of gilts may affect WMI in parity 1 sows.

Also, the relatively high ICC for herd variance indicates that there were large herd effects on the probability of parity 1 sows having WMI 4 days, likely because their herd management differed in terms of aspects such as hormonal treatments, heat detection programs, lactational feed intake and genetics.

Finally, there are some limitations that should be noted in this observational study performed using commercial herd data. For example, use of hormone treatments, health status, nutritional programs, pen groups and genotype were not taken into account in the analysis. Even with such limitations, this research provides valuable information for pig producers and veterinarians about WMI as a predictor for lifetime performance of sows.

## Conclusion

Recording WMI in parity 1 may help to predict a sow’s subsequent WMI and lifetime productivity. Also, parity 1 sows with WMI 4 or 5 days are likely to be fertile sows in breeding herds. Therefore, producers should adjust management to increase the proportion of parity 1 sows with WMI 4 or 5 days, aiming especially for WMI 4 days.

## Data Availability

The dataset analyzed during the current study is not publicly available because producers’ privacy could be compromised.

## Abbreviations

ICC: Intraclass correlation coefficients
WMI: Weaning-to-first-mating interval
